# Retrospective study of the outcomes of patients admitted to the ICU with a hematological malignancy

**DOI:** 10.1186/cc11016

**Published:** 2012-03-20

**Authors:** H Lewis, J Patel, N Lonsdale

**Affiliations:** 1 Birmingham Heartlands Hospital, Birmingham, UK

## Introduction

The UK prevalence of haematological malignancy is increasing. Seven percent of these patients become critically ill, necessitating ITU care [1]. The past decade has seen significant advances in the treatment and outcomes of patients with hematological malignancies. This has challenged the preconception that these patients are poor candidates for ICU admission. This study evaluated the trends in admission and outcomes of patients admitted to a general ICU with a diagnosis of hematological malignancy.

## Methods

A retrospective study of the last 50 consecutive admissions of patients with a hematological malignancy admitted to the ICU. Patients were identified from the ICNARC database. Demographic data, APACHE II, SOFA scores on admission, baseline neutrophil count and organ support data were collected. The primary outcome was ICU and hospital mortality. Data were compared against the cohort of patients admitted between April 2010 and April 2011.

## Results

The last 50 patients were admitted between January 2004 and August 2011. Overall the number of admissions increased throughout this period, with only one admission in 2004, peaking at 10 in 2009. In 2011, patients with a hematological malignancy represented 0.5% of all the ICU admissions. The commonest malignancies were acute myeloid leukemia (43%) and lymphoma (31%). The primary reason for admission was sepsis (61%), with pneumonia the commonest source (27%) and 42% admitted with neutropenic sepsis. Compared to the 2010/11 cohort the patients admitted with a hematological malignancy had significantly higher mean APACHE II scores (24 (SD 8) vs. 15 (SD 6) *P *< 0.0001), a longer mean ICU stay (10 days (SD 17) vs. 6 days (SD 10) *P *< 0.0001) and greater ICU (50% vs. 27% *P *< 0.0001) and hospital mortality (61% vs. 29% *P *< 0.0001). However, the overall trend was a considerable fall in mortality from 91% (2004 to 2007) to 36% (2008 to 2011). The mean SOFA score on admission for the hematological patients was 9 (SD 3). Twenty patients required two levels of organ support with only three patients receiving renal replacement therapy. No independent risk factors for outcome were identified.

## Conclusion

The outcomes of patients with hematological malignancies admitted to the ICU are improving with rates approaching that of our general ICU population. Patients with hematological malignancy requiring ICU admission continue to increase and admission should be based on their physiological derangement and overall prognosis. Further prospective studies are required to investigate potential predictors of outcomes in these patients.
